# 3D-Printed Janus Piezoelectric Patches for Sonodynamic Bacteria Elimination and Wound Healing

**DOI:** 10.34133/research.0022

**Published:** 2023-01-10

**Authors:** Danqing Huang, Yi Cheng, Guopu Chen, Yuanjin Zhao

**Affiliations:** ^1^ Institute of Translational Medicine, the Affiliated Drum Tower Hospital of Nanjing University Medical School, Nanjing 210002, China.; ^2^ State Key Laboratory of Bioelectronics, School of Biological Science and Medical Engineering, Southeast University, Nanjing 210096, China.; ^3^ Oujiang Laboratory (Zhejiang Lab for Regenerative Medicine, Vision and Brain Health), Wenzhou Institute, University of Chinese Academy of Sciences, Wenzhou, Zhejiang 325001, China.

## Abstract

Management of infected wounds has raised worldwide concerns. Attempts in this field focus on the development of intelligent patches for improving the wound healing. Here, inspired by the cocktail treatment and combinational therapy stratagem, we present a novel Janus piezoelectric hydrogel patch via 3-dimensional printing for sonodynamic bacteria elimination and wound healing. The top layer of the printed patch was poly(ethylene glycol) diacrylate hydrogel with gold-nanoparticle-decorated tetragonal barium titanate encapsulation, which realizes the ultrasound-triggered release of reactive oxygen species without leaking nanomaterials. The bottom layer is fabricated with methacrylate gelatin and carries growth factors for the cell proliferation and tissue reconstruction. Based on these features, we have demonstrated in vivo that the Janus piezoelectric hydrogel patch can exert substantial infection elimination activity under the excitation of ultrasound, and its sustained release of growth factors can promote tissue regeneration during wound management. These results indicated that the proposed Janus piezoelectric hydrogel patch had practical significance in sonodynamic infection alleviation and programmable wound healing for treating different clinical diseases.

## Introduction

Wounds have raised great concern in both the biomedical research field and clinical practice [1–4]. Infection is considered as a major factor in delaying the wound healing, which brings severe pain and heavy financial burden to patients. Thus, the healing process of the infected wounds usually includes pathogen elimination and tissue regeneration [5]. However, these 2 different but closely related pathological processes can hardly occur at the same time in the natural state, bringing about the hampered healing process. To address this problem, constant endeavors have been devoted to fabricating wound dressings such as paste and patch, encapsulating growth factors or antibacterial agents for the management of infected wounds [6–11]. Despite having positive curative efficacy, these clinically employed wound dressings often lack structural design and only completely cover the wound, which can seriously affect the oxygen exchange of the wound surface and impede tissue repair. Moreover, the uncontrolled release of these payloads cannot satisfy the diverse needs in different processes during wound healing. Therefore, a brand-new wound dressing patch with designed architectures and controllable drug release properties is met with great expectations for the management of infected wounds.

**Fig. 1.**
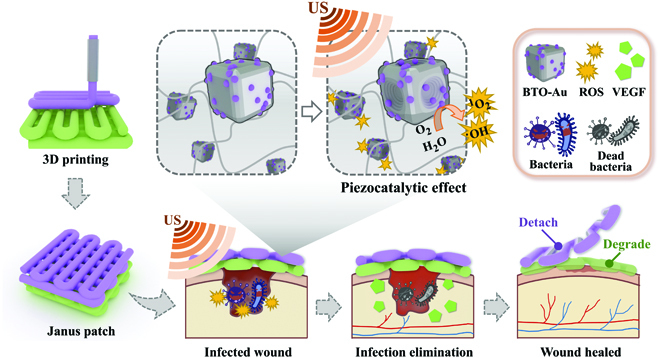
Schemes of the fabrication and application of the Janus patch. The piezoelectric materials and growth-factor-coloaded Janus hydrogel patch realized the US-excited bacteria elimination and promoted wound healing.

In this paper, inspired by the cocktail treatment and combinational therapy, we proposed 3-dimensional (3D)-printed Janus piezoelectric patches for sonodynamic bacteria elimination and wound healing, as shown in Fig. 1. Since the advancement of 3D printing technology, it has been applied for diverse practical applications in biomedical fields [12–19]. Owing to its manual controllability, 3D printing can realize the design and construction of fine structures. Inspired by the flexibility and air permeability of clinical gauzes, it is assumed that a 3D-printed hydrogel patch with a mesh design would be practical for wound dressing. In contrast, sonodynamic treatments based on the ultrasound (US)-sensitive materials have shown considerable advantages in fighting pathogens [20–25]. Among the US-sensitive materials, piezoelectric nanomaterials have been elucidated to produce reactive oxygen species (ROS) effectively [1,24,26–28]. Contrary to traditional sonosensitizers that were derived from photosensitizers, piezoelectric nanomaterials, such as gold-nanoparticle-decorated tetragonal barium titanates (BTO-Au), feature reduced adverse effects and improved biocompatibility [1,29]. Despite having good biological effects, the poor maneuverability and uncertain clearance of these nanomaterials remain a limitation during applications. Thus, it is conceived that a 3D-printed multicompartment patch with spatial encapsulation of piezoelectric nanomaterials would eliminate the disadvantages of nanomaterials and improve wound prognosis simultaneously.

Herein, we fabricated a Janus hydrogel patch via 3D printing for sonodynamic bacterial elimination and wound healing. Janus materials refer to a composite structure with 2 sides of different properties. The top layer fabricated with poly(ethylene glycol) diacrylate (PEGDA) was designed to load piezoelectric nanocomposites, while the bottom layer fabricated with methacrylate gelatin (GelMA) was used to load vascular endothelial growth factor (VEGF). The 3D-printed Janus patch showed desired physical integrity and controllable drug release kinetics. Owing to the mechanical strength of PEGDA, the piezocatalytically generated ROS can be released from the top layer and eliminate bacteria effectively, without releasing the encapsulated BTO-Au nanomaterials. In contrast, the inherent bioactivity of the bottom GelMA can support cell adhesion and proliferation, the sustained release of VEGF can promote the angiogenesis in the wound healing process, and the biodegradability of GelMA at the wound site can avoid the removal of patches. In the mouse model with infected wound, we customized the shape of the hydrogel patch according to the dorsal wound, realizing the even distribution and aggregation of the loaded drugs. Additionally, the mesh design ensured the oxygen supply during angiogenesis. These features revealed that the multicompartment Janus patch had practical significance in fighting bacterial infection and subsequently promoting tissue regeneration, shedding light on the combination between sonodynamic inflammation alleviation and programmable wound management.

## Results

**Fig. 2.**
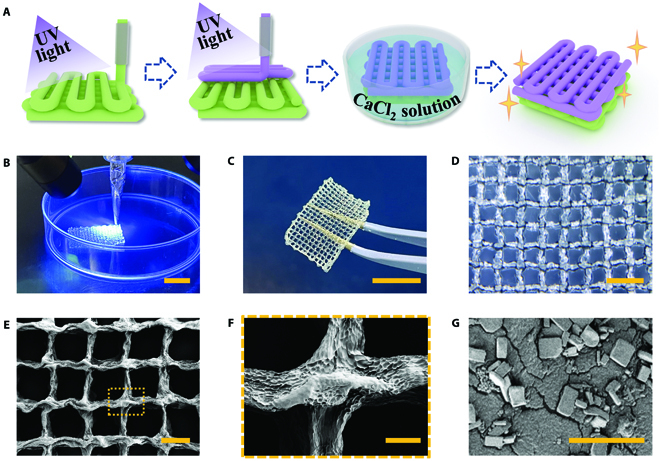
Fabrication and characterization of the 3D-printed Janus hydrogel patch. (A) Schematic of the fabrication process of the Janus patch. (B) Real-time image of the UV-assisted 3D printing process. (C) Digital image of a hydrogel patch. (D) Optical image of the hydrogel patch. (E to G) SEM images of the hydrogel patch (E), the magnified view of the hydrogel fiber cross (F), and BTO-Au nanocomposites inside the hydrogel fiber (G). The scale bars are 1 cm in (B) and (C), 2 mm in (D), 1 mm in (E), 200 μm in (F), and 20 μm in (G).

In a typical experiment, an extrusion-based 3D printer driven by pneumatic force was employed for the layer-by-layer fabrication of the Janus hydrogel patch. The bottom layer was first printed by extruding a mixture of VEGF-contained GelMA pregel and alginate from a syringe, accompanied by continuous ultraviolet (UV) light exposure. The alginate with high concentration contributed to the consistency of the bioink, promoting the maintenance of the preliminary shape. After the formation of the first layer, another syringe containing a mixture of BTO-Au nanocomposites, PEGDA pregel, and alginate was employed to print the top layer. In addition to the photo-crosslinking of GelMA and PEGDA by UV light, the whole hydrogel patch was then immersed into the calcium chloride (CaCl_2_) solution to ionically crosslink alginate and Ca^2+^, enhancing the mechanical strength and stability of the mesh structure (Fig. 2A). Figure 2B shows the 3D printing process of the hydrogel patch, indicating that the formulations had good printability and shape consistency. As illustrated in Fig. 2C and D, the finally obtained hydrogel patch featured excellent mechanical integrity and stable mesh-designed structures. The scanning electron microscope (SEM) images of the patch exhibited the desired shape retention of the 3D-printed hydrogel (Fig. 2E and F), implying its stability during practical applicability. The SEM image of the cross section of the hydrogel fiber showed the existence of BTO-Au, which were presented as cubic nanomaterials (Fig. 2G). The degradation characteristics of the GelMA patch and the PEGDA patch were also measured, and the results suggested that the better degradability of GelMA enabled the separability of the PEGDA patch (Fig. S1).

**Fig. 3.**
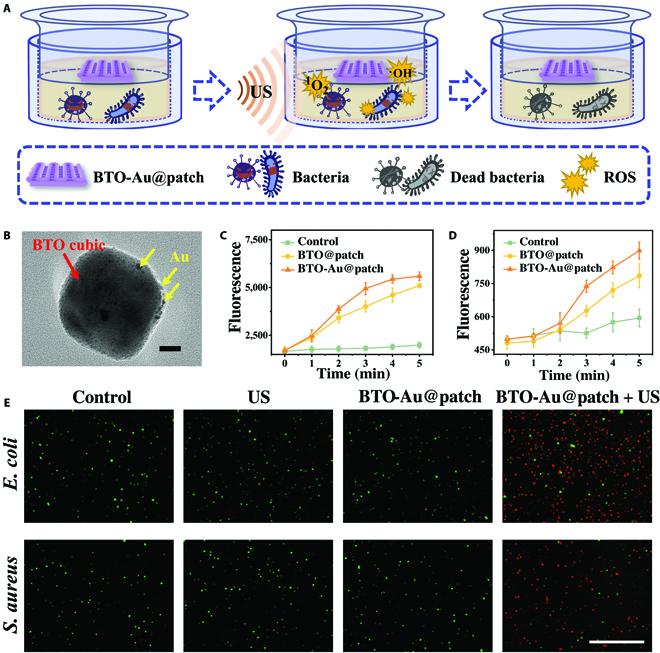
The US-triggered piezoelectric ROS generation and sonodynamic antibacterial efficacy of the BTO-Au@patch. (A) Schemes of the sonodynamic antibacterial treatment in vitro. (B) TEM image of BTO-Au nanocomposites. Red arrow indicates the BTO nanocubic, and yellow arrows indicate the Au nanoparticles. (C and D) The fluorescence intensity of SOSG (C) and HTA (D) after different times of US irradiation. (E) Live and dead staining fluorescent images of *E. coli* and *S. aureus* in different groups. The scale bars are 20 nm in (B) and 100 μm in (E).

Then, we verified that the piezoelectric ROS production of PEGDA-encapsulated BTO-Au could exert a sonodynamic antibacterial effect (Fig. 3A). From the transmission electron microscope (TEM) image, Au nanoparticles were found to be randomly attached to the surface of the tetragonal barium titanate (BTO) nanocrystal, which suggested the successful fabrication of BTO-Au (Fig. 3B). Recently, the tetragonal BTO nanocrystals have been elucidated to feature an excellent piezoelectric coefficient [1,24,26,28]. Under the excitation of the periodic ultrasonic vibration, mechanical deformation and piezoelectricity occurred on the tetragonal BTO. Then, the electron–hole pairs on the BTO are separated and lead to the establishment of a built-in electric field, followed by the production of ROS via redox reactions [30]. Additionally, the Schottky junction between Au particles and BTO nanocrystals could induce the band bending of BTO, promoting the separation of electron–hole pairs, thus further enhancing the production of ROS (Fig. S2) [29]. As reported by various researchers, the US-induced piezocatalytic ROS production could significantly contribute to the elimination of pathogens without unwanted effects [31,32]. Moreover, in another research proposed by Zhu et al. [26], it was proved that encapsulating BTO nanoparticles into thermogels would not affect their piezoelectricity; they still showed excellent tumor-killing efficacy against 4T1 cells. Thus, based on the abovementioned research results, we assumed that our BTO-Au-encapsulated PEGDA hydrogel patch (BTO-Au@patch) was promised to display desired US-induced piezocatalytic effects.

We then verified that the PEGDA hydrogel entrapment would not weaken the piezocatalytic efficiency of BTO-Au. First, we applied the singlet oxygen sensor green (SOSG) to measure the generation of singlet oxygen (^1^O_2_). As a commercial ROS probe, SOSG would emit high fluorescence when interacted with ^1^O_2_ in vitro. The empty-loaded PEGDA hydrogel patch was set as control, and the PEGDA hydrogel patch encapsulating BTO or BTO-Au was set as the BTO@patch group and the BTO-Au@patch group, respectively. In the presence of BTO and BTO-Au, the fluorescence intensity of SOSG at 522 nm elevated gradually with the extension of US irradiation time, indicating the time dependency of US-triggered piezocatalysis (Fig. 3C). Compared with the BTO@patch group, the ^1^O_2_ production after US excitation in the BTO-Au@patch group was significantly higher. Next, to detect the hydroxyl radical (•OH) generation, we applied terephthalic acid (TA) as a probing molecule. It has been proven that the reaction between TA and •OH could produce 2-hydro-xyterephthalic acid (HTA), the fluorescence intensity of which can be detected at 426 nm. As illustrated in Fig. 3D, the •OH generation of the BTO-Au@patch was 1.14 times higher than that of the BTO@patch, implying the significantly enhanced •OH producibility of BTO-Au. These results demonstrated the time-dependent ROS generation of US-excited BTO-Au and that the PEGDA entrapment of BTO-Au would not affect its ROS producibility.

The antibacterial activity of US-excited BTO-Au@patch was elucidated on *Staphylococcus aureus* (*S. aureus*) and *Escherichia coli* (*E. coli*), representing the Gram-positive and -negative bacteria, respectively. The bacteria were divided into 4 groups randomly, namely, control group, US group, BTO-Au@patch group, and BTO-Au@patch + US group. For the US group, 1.5 W cm^−2^ intensity power and 1.0 MHz central frequency ultrasonic wave was applied to irradiate the bacteria for 3 min. For the BTO-Au@patch and the BTO-Au@patch + US group, a piece of the BTO-Au-loaded PEGDA patch was placed onto the transwell of the plate, followed by 3 min of US irradiation to the BTO-Au@patch + US group. After 3 h of incubation, the bacteria in each group were stained with Calcein-AM/PI for further evaluation. As illustrated in Fig. 3E, compared with the control group, the US group and the BTO-Au@patch group did not exhibit obvious discrepancy, indicating that simple US irradiation and hydrogel-encapsulated BTO-Au would not harm the bacteria significantly. In contrast, large amounts of dead bacteria could be observed in the BTO-Au@patch + US group. Furthermore, the quantification of antibacterial experiments also verified the US-activated piezocatalytic effect against *S. aureus* and *E. coli* (Fig. S3). Therefore, the BTO-Au patch featured promising antibacterial efficacy, which might contribute to the treatment of infected wound.

**Fig. 4.**
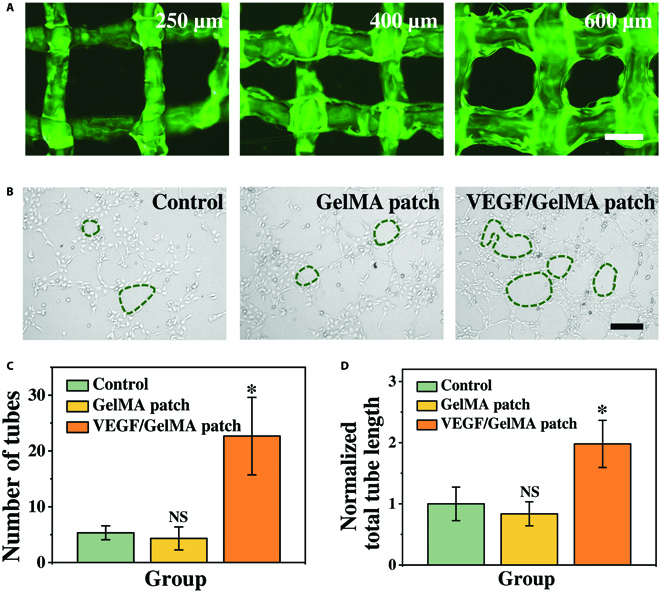
The angiogenetic effects of the VEGF-loaded GelMA/alginate patch. (A) The fluorescent images of the FITC-BSA-loaded GelMA/alginate patch with a line width of 250, 400, and 600 μm. (B) The representative optical microscopic images of HUVECs cultured in different groups. (C and D) Statistical analysis of the number of capillary-like structures (C) and the normalized total tube length (D) from different groups. The scale bars are 500 μm in (A) and 200 μm in (B).

In addition to the sonodynamic antibacterial effect of the BTO-Au@patch, the VEGF-loaded GelMA patch (VEGF-patch) is supposed to play a dominant role in promoting tissue reconstruction and wound healing. Thus, we explored the drug release kinetics and the angiogenetic effects of the 3D-printed VEGF/GelMA patch. Owing to the customizability and maneuverability of 3D printing, we fabricated fluorescein isothiocyanate-labeled bovine serum albumin (FITC-BSA)-encapsulated mesh-structured hydrogel patches with line widths of 250, 400, and 600 μm to investigate the drug release activities (Fig. 4A). As demonstrated in Fig. S4, the release of protein molecules was a long-term process. According to the curve trend, the sustained release of the payloads was required during the process of tissue regeneration. The results illustrated that the hydrogel patches with a smaller line width (250 μm) had relatively higher release percentage. However, the fine lines did not have enough mechanical strength to maintain the physical integrity, as partial fraction of the mesh structure could be observed in Fig. 4A. However, since the relatively low release percentage of hydrogel patches with a larger line width (600 μm) would lead to a waste of drugs, a line width of 400 μm was optimized for downstream experiments. In addition, the mechanical integrity and stability of a patch with a 400-μm line width was confirmed after being bent, distorted, and immersed underwater (Fig. S5), implying its stability during biomedical applications.

VEGFs are strong mitogens for promoting the migration of endothelial cells and formation of blood vessels. In order to investigate the proangiogenesis effect of the VEGF-patch, we applied human umbilical vein endothelial cells (HUVECs) to perform the tube formation study. As shown in Fig. 4B, the HUVECs cultured in blank medium (control group) and medium containing the unloaded GelMA hydrogel patch (GelMA patch group) developed few capillary-like structures. In contrast, the HUVECs cultured with the VEGF-patch could be observed to develop numerous tight cell junctions and intact tube structures. Statistical analyses also displayed the significantly increased number of capillary-like structures of HUVECs cultured with the VEGF-patch (Fig. 4C). The normalized total tube lengths of HUVECs in the VEGF-patch group were also significantly higher than those in the control and the GelMA patch group (Fig. 4D). First, the results of the tube formation studies indicated the biocompatibility of the 3D-printed hydrogel patch. Most importantly, the GelMA-encapsulated VEGFs were proved to maintain excellent bioactivity after undergoing the 3D printing process and being released from the GelMA hydrogel fibers, which suggested the mild fabrication process and the biofavorability of GelMA. These above-mentioned studies illustrated that the multicompartment Janus hydrogel patch with the BTO-Au-loaded PEGDA top layer and the VEGF-loaded GelMA bottom layer had a promising potential to exert favorable effects in promoting antibacterial wound healing.

**Fig. 5.**
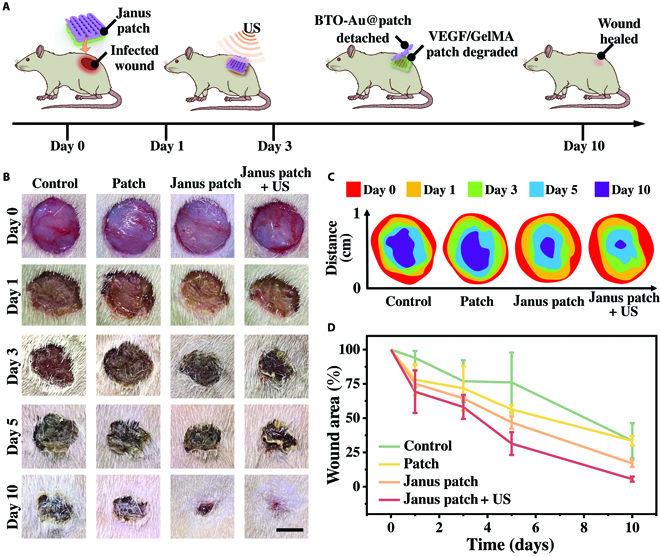
The sonodynamic antibacterial activity on the rats with infected wound. (A) Schematic diagram of the application of the Janus patch and sonodynamic antibacterial treatment. (B) Representative images of the dorsal wounds treated with PBS, empty-loaded patch, Janus patch, and Janus patch accompanied with US irradiation from day 0 to day 10. (C) Size of the wound closure area from different groups. (D) The relative wound area with different treatments from day 0 to day 10. The scale bar is 5 mm in (B).

Wound healing is a multistage process, especially for the infected wounds. The inflammation caused by pathogens would obstruct the normal cells from absorbing nutrition and oxygen, thus hindering the neovascularization and tissue regeneration. Encouraged by the positive results of the above-mentioned in vitro experiments, we conducted in vivo experiments to evaluate the antibacterial efficacy and proangiogenesis activity of the multicompartment hydrogel gauze. After the establishment of a dorsal full-thickness skin wound, the *S. aureus* suspension was added to the wound to build an infected model. The rats were randomly divided into 4 groups, namely, the control group, the empty-loaded gauze group, the Janus patch group, and the Janus patch + US group (Fig. 5A and Fig. S6). During the wound healing and treatment process, the rats maintained a healthy state and grew steadily, indicating the biocompatibility of the hydrogel patches and US irradiation (Fig. S7). Generally, the wound area of all groups decreases from day 0 to day 10 (Fig. 5B). The wounds in the Janus patch + US group presented the best outcome, followed by the Janus patch group. In contrast, the rats in the control group and the empty-loaded patch group had the slowest wound closure rate with apparent scars (Fig. 5C and D and Fig. S8). Notably, although the wound healing process in the Janus patch group promoted the sustained release of VEGF from the GelMA layer, the delayed infection elimination hindered the wound closure. Characteristics of the morphological changes of wounds from different groups implied the substantial role of sonodynamic antibacterial activity in promoting wound healing.

**Fig. 6.**
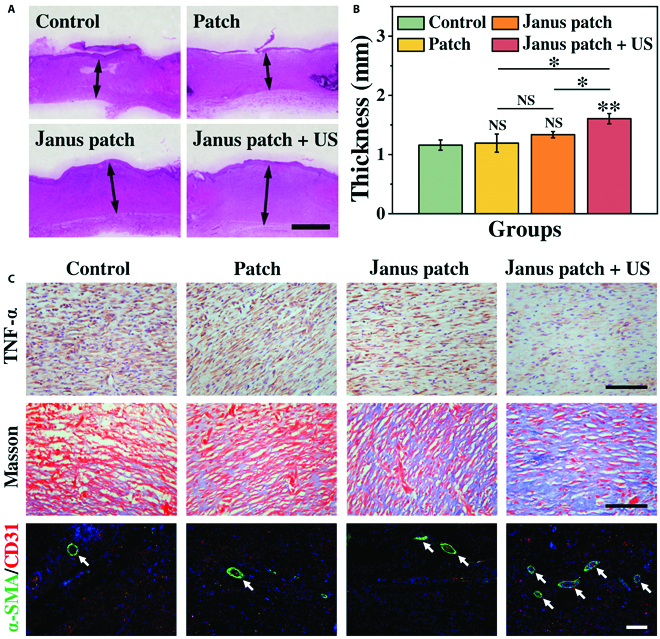
Histological analysis of the wound status. (A) H&E staining of the wound tissue. (B) Statistical analysis of the granulation tissue thickness. (C) Masson staining, immunostaining of TNF-α, and double staining of alpha smooth muscle actin (α-SMA) and CD31 of tissue samples from different groups. The scale bars are 1 mm in (A) and 100 μm in (C).

To analyze the tissue regeneration after different treatments, hematoxylin-eosin (H&E) staining was carried out to investigate the granulations of the wounds (Fig. 6A). By measuring the thicknesses of the granulation tissues, we found that the granulation tissues in the control group and empty-loaded patch group were relatively thinner, while the granulation tissues in the Janus patch + US group were significantly thicker, which was an ideal outcome for wound healing (Fig. 6B). Through the immunostaining of tumor necrosis factor-α (TNF-α) from different groups, large amounts of TNF-α can be observed in the control, patch, and Janus patch groups, indicating a severe inflammatory response in wound area (Fig. 6C). However, significantly decreased TNF-α expression can be found in the Janus patch + US group, which suggested the effective sonodynamic antibacterial effect of the US-excited BTO-Au@patch. To further analyze the tissue reconstruction efficacy of the Janus patch, Masson staining was carried out on different groups. Compared with the control and patch group, significantly increased collagen deposition can be detected in the Janus patch and Janus patch + US group, which indicated the neovascularization efficacy of the VEGF-patch and the mesh-structure-affiliated oxygen exchange [33]. Since angiogenesis plays a critical role in tissue maturation, we further investigate the vessel formation in different groups through the double immunostaining of alpha smooth muscle actin and CD31. Large amounts of vascular structures can be found in the Janus patch + US group, while the vascular formation was hardly detected in the control and patch group (Fig. S9). These results elucidated the effective sonodynamic antibacterial efficacy and angiogenic property of the US-accompanied Janus patch.

## Discussion

In summary, we fabricated a Janus hydrogel patch with mesh structures for programmable antibacterial wound healing. Owing to the good printability of GelMA and PEGDA, the 3D-printed hydrogel patch showed excellent shape consistency and physical integrity. By encapsulating the piezoelectric nanomaterials BTO-Au into PEGDA/alginate hydrogel, we realized the high aggregation and accumulation of BTO-Au in the infected wound region and targeted the release of the US-excited piezocatalytic ROS, avoiding leakage of nanomaterials. Notably, compared to simple BTO nanocubes, the Au-decorated BTO nanocubes showed significantly enhanced ROS production, leading to a better antibacterial efficacy. Subsequently, the internal bioactivity of GelMA hydrogel along with the consistent release of VEGF contributed to cell proliferation and tissue regeneration after eliminating the infection. In the mouse model with infected dorsal wounds, it was demonstrated that the multicompartment mesh-structured Janus patch could efficiently alleviate infection and promote angiogenesis. Additionally, the biodegradable GelMA patch provided a scaffold for tissue reconstruction and enabled the separation of the BTO-Au@patch, avoiding the secondary damage to the healing wound caused by the removal of wound dressing and addressing the post-treatment management of nanomaterials. Thus, our 3D-printed Janus hydrogel patch could be used as a reliable synergistic drug delivery system for personalized and programmable wound management.

## Materials and Methods

### Materials, cell lines, and animals

Sodium alginate, PEGDA, calcium chloride, ammonia, gelatin, methacrylic acid, and *N*,*N*-dimethyl-*p*-phenylenediamine sulfate (HMPP) were obtained from Sigma-Aldrich. GelMA was synthesized from gelatin and methacrylic acid in the laboratory. BTO was obtained from Yu-Mu Materials. Hydrogen tetrachloroaurate trihydrate and TA were obtained from Energy Chemicals. M199 was obtained from Jiangsu KeyGEN BioTECH Corp. Fetal bovine serum, penicillin/streptomycin, trypsin-EDTA solution, and phosphate-buffered saline (PBS) were purchased from Gibco. Growth factor reduced Matrigel was obtained from BD Biosciences. SOSG was purchased from Invitrogen. The Calcein-AM/PI staining kit was purchased from Dojindo Molecular Technologies. HUVECs were obtained from the Cell Bank of the Chinese Academy of Sciences. The Sprague–Dawley rats weighed 200 g and were obtained from the Model Animals Research Center of Nanjing University. All animal experiments were performed according to guidelines set by the Animal Ethics Committee of Drum Tower Hospital affiliated to the Medical School of Nanjing University (No. 2020AE01054).

### Fabrication of Au-decorated BTO

BTO nanocubes (50 mM) were mixed with 10 ml of hydrogen tetrachloroaurate trihydrate in an alkaline environment created by adding NH_3_·H_2_O (pH 11.0). The Au^3+^ concentration in the final solution was 10 mg ml^−1^. The mixture was stirred at 75 °C for 2 h. Then, the mixture was centrifuged and washed with deionized water 3 times, followed by calcining at 300 °C for 1 h. The morphology of BTO-Au was characterized by an SEM (HITACHI, S-3000N) and a TEM (JOEL, JEM-2100).

### Preparation of hydrogel bioink

For the top layer, sodium alginate (15 wt.%), PEGDA (15 wt.%), and HMPP (0.5 wt.%) were mixed and stirred overnight in the dark, with or without adding BTO-Au (1 mM). For the bottom layer, sodium alginate (15 wt.%), GelMA (10 wt.%), and HMPP (0.5 wt.%) were mixed and stirred overnight in the dark, with or without adding VEGF (0.1 wt.%). All operations were carried out under dark conditions. The mixed formulations were placed into the syringe (syringe 1 contained the PEGDA formulation, and syringe 2 contained the GelMA formulation) and kept at 4 °C to improve the viscosity.

### Fabrication and characterization of the Janus patch

A cubic scaffold with patch-like structures was designed for 3D printing (20.00 mm length × 10.00 mm width × 2.00 mm height), and a nozzle with an inner diameter of 300 μm was employed. The settings of the 3D printer (Bio-Architect, Regenovo) were as follows: a printing pressure of 0.15 MPa, a printing speed of 4 mm/s, and a printing temperature of 22 °C. Syringe 2 was first employed to print the bottom layer. After the 2 layers of interlaced hydrogel fibers, syringe 1 was employed to print the top layer. During the 3D printing process, the printed hydrogel was exposed by UV light from the side. After printing, the primary hydrogel patch was immersed into 2 wt.% CaCl_2_ for 1 h. Then, the hydrogel patch was washed 3 times with PBS. A stereomicroscope (JSZ6S, Jiangnan Novel Optics) was first used to observe the physical integrity and shape consistency of the hydrogel patch. The microstructures of the hydrogel patch and the encapsulated BTO-Au were characterized by an SEM.

### Detection of the piezoelectric ROS generation

A therapeutic ultrasonic wave (an intensity power of 1.5 W cm^−2^, a central frequency of 1.0 MHz, and a duty cycle of 50%) (Sonoplus 190, Enarf-Nonius, Holland) was applied to excite the piezoelectric effect. To measure the ^1^O_2_ generation, the empty-, BTO-, and BTO-Au-loaded hydrogel patches (1 mM BTO/BTO-Au) were immersed in 2 ml of deionized water in centrifuge tubes, and then the US transducer was applied to the lateral wall. At different time intervals, 100 μl of sample was extracted and added in a 96-well plate, followed by mixing with the SOSG solution (40 mg l^−1^). The fluorescence intensity of SOSG was detected with an excitation wavelength at 504 nm and an emission wavelength at 522 nm. To measure the •OH generation, the empty-, BTO-, and BTO-Au-loaded hydrogel patches were immersed in 2 ml of TA solution (0.8 mg l^−1^ NaOH and 1.0 mg l^−1^ TA). After various treatments of US, 100 μl of sample was extracted and added in a 96-well plate. The fluorescence intensity of HTA was detected with an excitation wavelength at 315 nm and an emission wavelength at 426 nm.

### In vitro antibacterial tests

The Gram-positive bacteria *S. aureus* and Gram-negative bacteria *E. coli* were selected as representative bacteria for investigating the antibacterial activity of the piezocatalytic effect. Typically, *S. aureus* and *E. coli* (2 × 10^5^ CFU ml^−1^) were seeded into 6-well plates. For the BTO-Au@patch group and the BTO-Au@patch +US group, a transwell loaded with the BTO-Au PEGDA patch was placed into the plate in order to reduce the deviation of results. For the BTO-Au@patch +US group, the US transducer (1.5 W cm^−2^, 1.0 MHz, 50% duty cycle) was applied to the lateral wall of plate for 3 min. After 3 h of incubation, the *S. aureus* and *E. coli* were stained with Calcein-AM/PI and a fluorescence microscope (Carl Zeiss, Germany) was utilized to observe the viability of cells. The live bacteria were represented as green fluorescence, and the dead bacteria were represented as red fluorescence.

### Measurement of the drug release

FITC-BSA (0.01 mg l^−1^) was employed to simulate the release of VEGF in the GelMA patch. First, the FITC-BSA-loaded GelMA patch was fabricated by printing bioink containing sodium alginate (15 wt.%), GelMA (10 wt.%), HMPP (0.5 wt.%), and FITC-BSA, followed by UV irradiation and Ca^2+^ ionic crosslinking. By regulating the printing speed and nozzle size, patches with different line widths can be obtained. A small patch (5.00 mm length × 5.00 mm width) was cut off from the original patch and immersed into the PBS at 37 °C. Then, 100 μl of sample was extracted at certain time intervals. The release amount of FITC-BSA was quantified according to the standard curve.

### Tube formation assay

HUVECs cultured in M199 medium with 10% fetal bovine serum were employed to analyze the bioactivity and release kinetics of the VEGF-loaded GelMA/alginate patch. After the spread plate operation and 37 °C gelation of 100 μl of Matrigel in a 24-well plate, 2 × 10^5^ HUVECs ml^−1^ were seeded in each well. Blank medium, medium containing the empty-loaded hydrogel patch, and medium containing the VEGF-loaded hydrogel patch (0.1 wt.% VEGF) was added to each well. After 12 h of culture, an optical microscope was used to record the capillary-like tube formation.

### Establishment of the infected wound model

The Sprague–Dawley rats were anesthetized, followed by the removal of a 1-cm-diameter round full-thickness skin on the back. Then, 200 μl of *S. aureus* suspension (2 × 10^6^ CFU ml^−1^) was added onto the wound. In the control group, the wounds were treated with PBS. In the second group, the wounds were covered with round empty-loaded patches. In the third and fourth group, the wounds were covered with BTO-Au and VEGF-coloaded Janus patches. In the fourth group, the wounds were treated with 3 min of US irradiation in the first 3 days and then the BTO-Au@PEGDA patch was removed. The wounds were moist surfaces with affinity for the hydrophilic hydrogel patch, and a small amount of sterile ultrasonic coupler was applied to the wound to facilitate US treatment. The wounds in each group were photographed on days 0, 1, 3, 5, and 10. After the 10-day treatment, the rats were weighted and sacrificed and the granulation tissues were collected for histology and immunohistochemistry analysis.

### Statistical analysis

The Origin 2019b software was applied to analyze data. The discrepancies between groups were calculated using 2-sided Student’s *t* test. When *P* value was less than 0.05, the results were defined as statistically significant; * represents *P* < 0.05, ** represents *P* < 0.01, *** represents *P* < 0.001, and NS represents not significant.

## Data Availability

The data that support the findings of this study are available in the supplementary materials of this article.

## Supplementary Materials

Supplementary Materials Fig. S1. Characterization of the Janus patch. Fig. S2. ROS producibility of US-excited BTO and BTO-Au. Fig. S3. Statistical analysis of the live/dead staining of (A) *E. coli* and (B) *S. aureus* from different groups. Fig. S4. Statistical analysis of the FITC release from patches with different widths. Fig. S5. No crack or scatter was observed on the hydrogel patch with a 400-μm line width after being (A) bent, (B) distorted, and (C) immersed underwater. Fig. S6. Different treatments of wounds. Fig. S7. Statistical analysis of the rat weight from different groups. Fig. S8. Statistical discrepancies of the wound areas on day 10. Fig. S9. Quantitative study of density of blood vessels from different groups on day 10.
