# Supplementary material for: 3D-Printed Janus Piezoelectric Patches for Sonodynamic Bacteria Elimination and Wound Healing
Source: Research (Wash D C). 2023 Jan 10;6:0022. doi: 10.34133/research.0022 (PMC10076028; doi:10.34133/research.0022)
Supplement: Supplementary Materials — Fig. S1. Characterization of the Janus patch. Fig. S2. ROS producibility of US-excited BTO and BTO-Au. Fig. S3. Statistical analysis of the live/dead staining of (A) E. coli and (B) S. aureus from different groups. Fig. S4. Statistical analysis of the FITC release from patches with different widths. Fig. S5. No crack or scatter was observed on the hydrogel patch with a 400-μm line width after being (A) bent, (B) distorted, and (C) immersed underwater. Fig. S6. Different treatments of wounds. Fig. S7. Statistical analysis of the rat weight from different groups. Fig. S8. Statistical discrepancies of the wound areas on day 10. Fig. S9. Quantitative study of density of blood vessels from different groups on day 10. [file research.0022.f1.docx]

Supplementary Materials


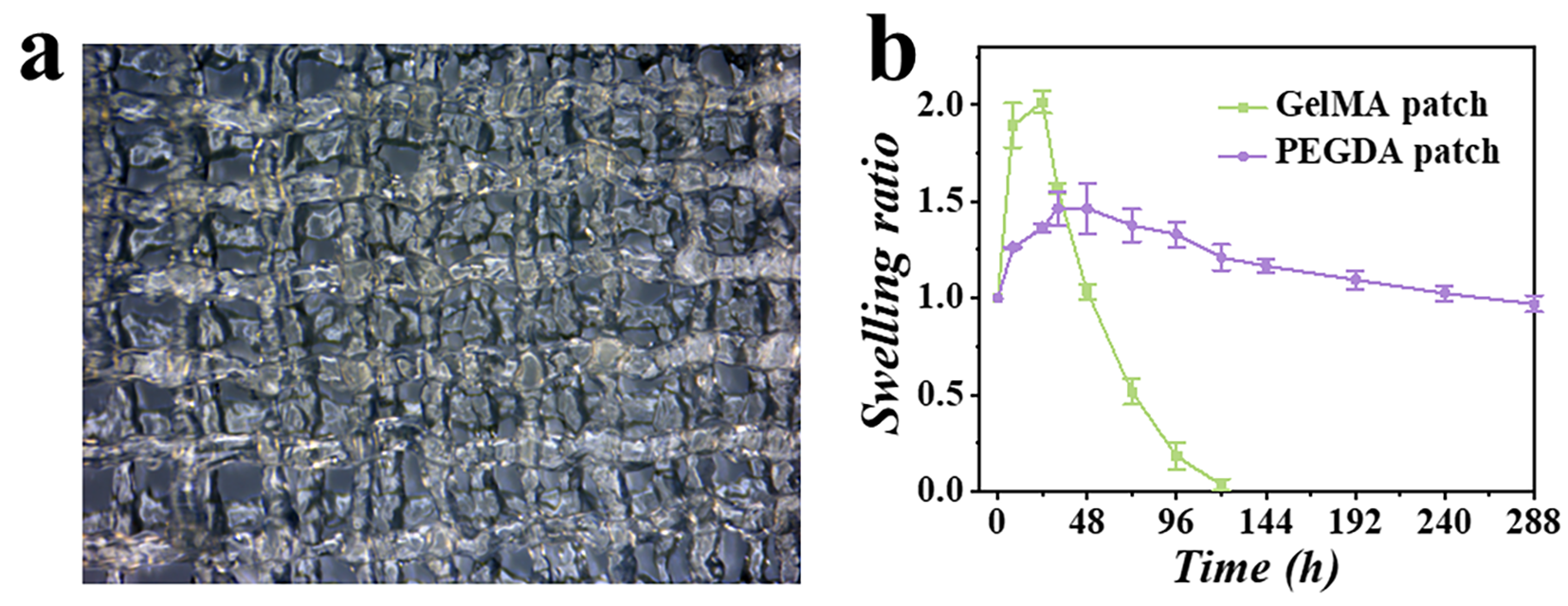


Figure S1. Characterization of the Janus patch. (a) Stereomicroscopic image of the Janus patch. (b) The swelling ratio and degradation analysis of the GelMA patch and PEGDA patch.


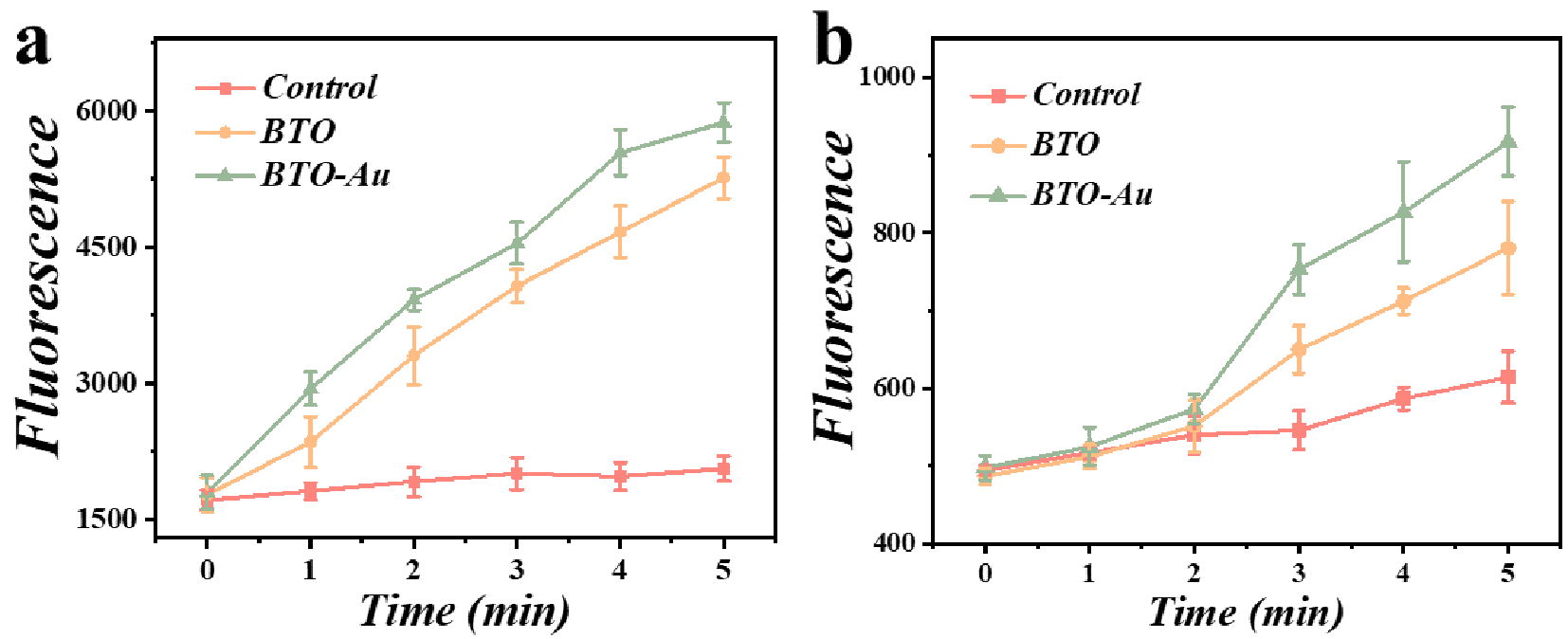


Figure S2. ROS producibility of US-excited BTO and BTO-Au.


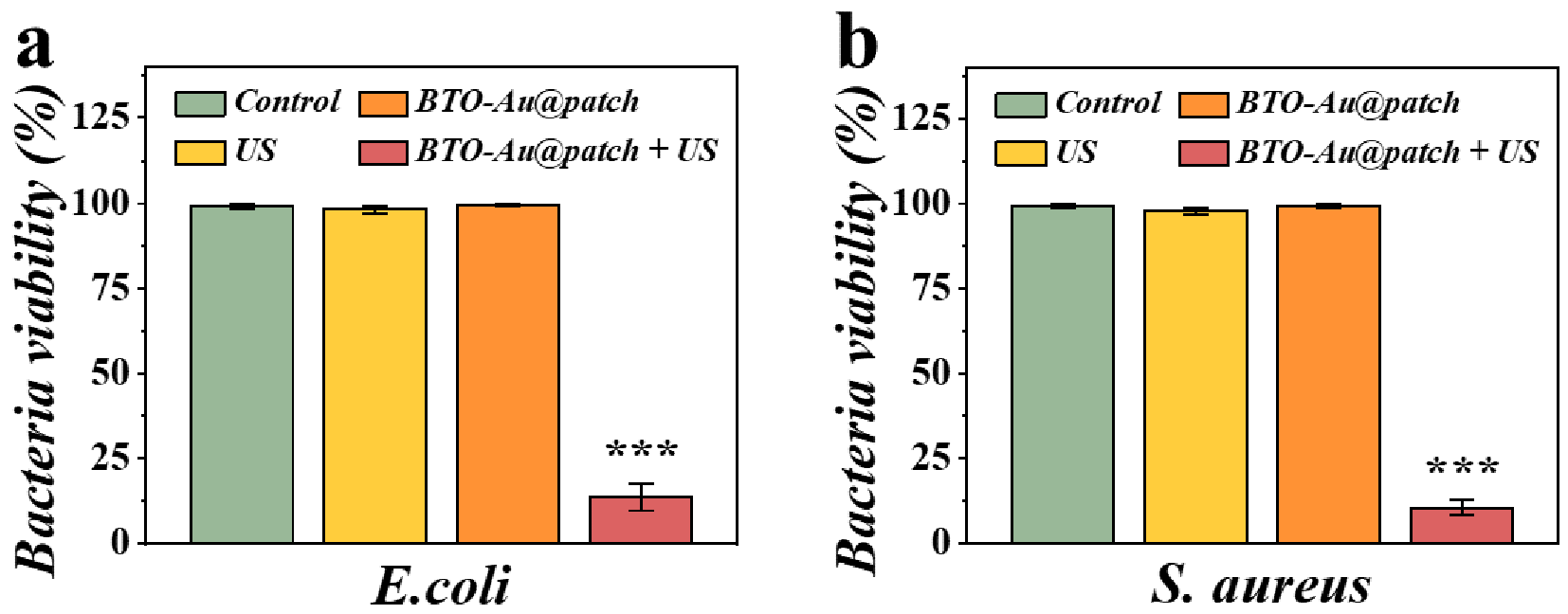


Figure S3. Statistical analysis of the live/dead staining of (a) E. coli and (b) S. aureus from different groups.


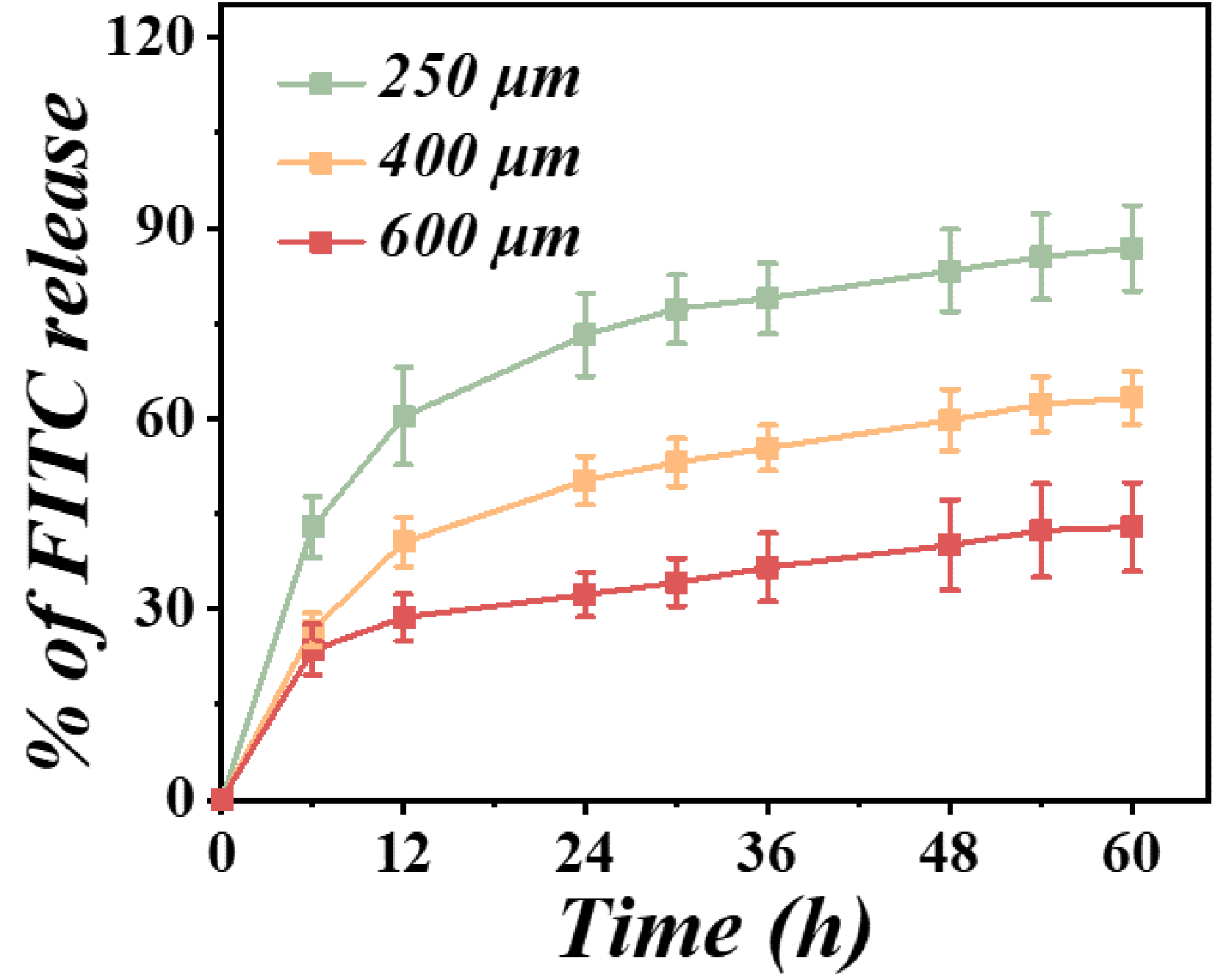


Figure S4. Statistical analysis of the FITC release from patches with different widths.


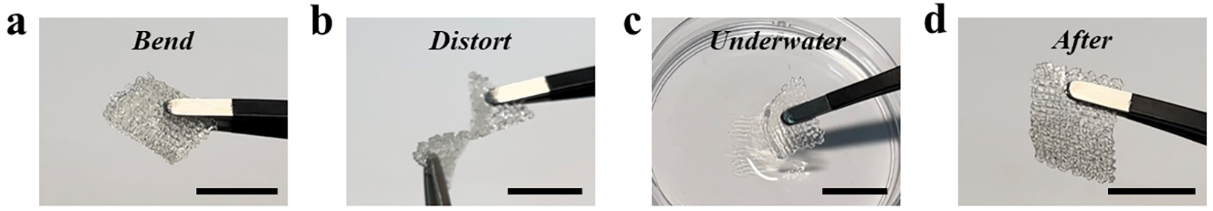


Figure S5. No crack or scatter was observed on the hydrogel patch with 400 μm line width after being (a) bent, (b) distorted, and (c) immersed underwater. (d) The shape and structure remained intact. Scale bar represents 1 cm.


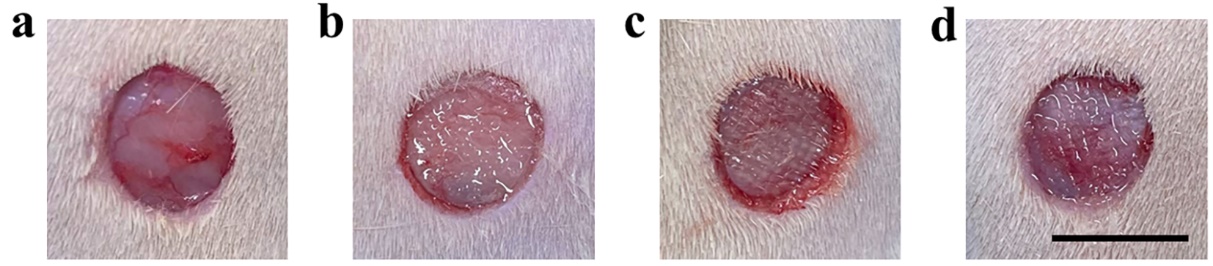


Figure S6. Different treatments of wounds. (a) Control group treated with normal saline. (b) Empty-loaded patch group was treated with GelMA/PEGDA patch. (c) Janus patch group was treated with Janus patch. (d) Janus patch + US group was treated with Janus patch along with US irradiation. Scale bar represents 1 cm.


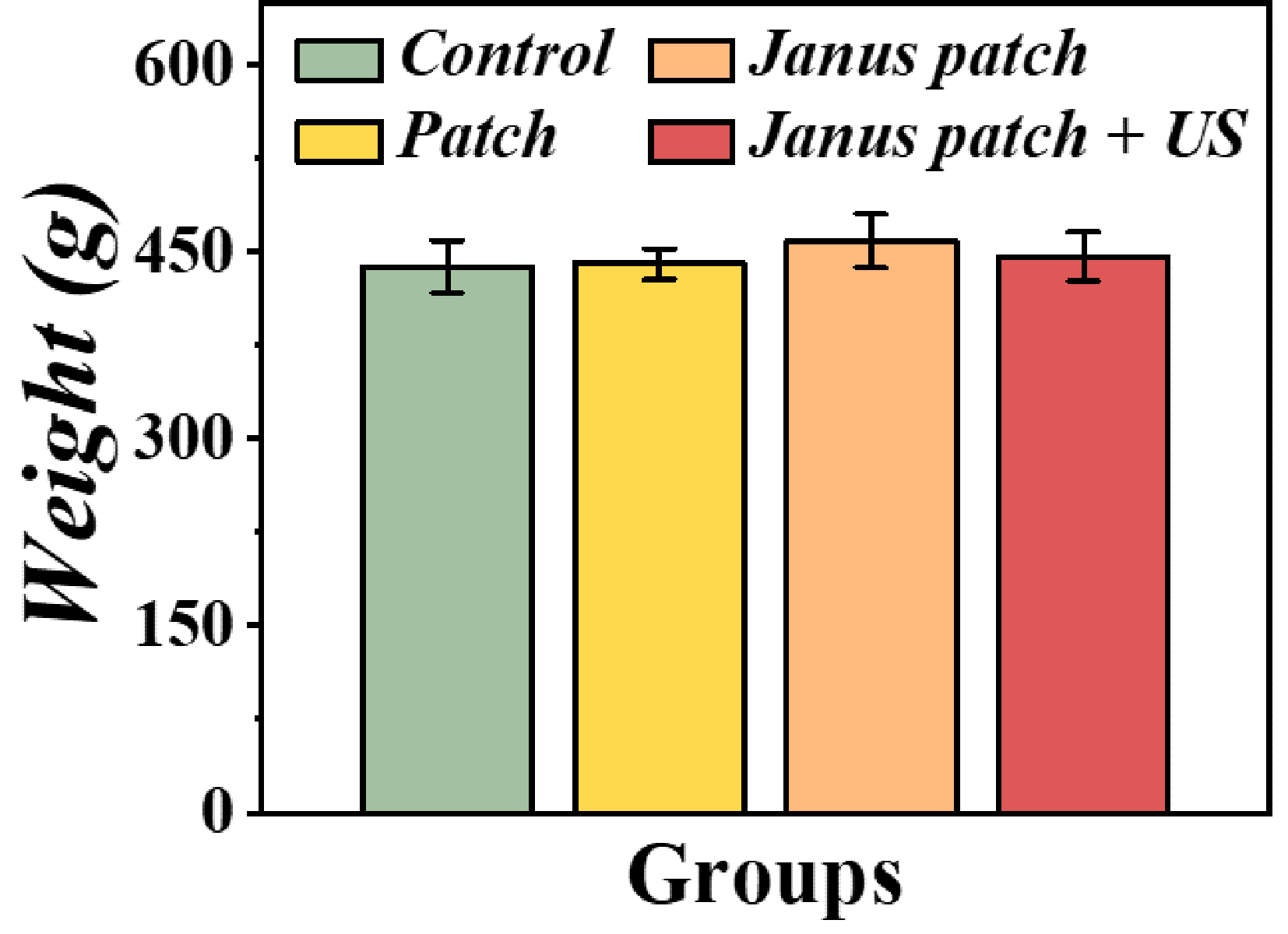


Figure S7. Statistical analysis of the rat weight from different groups.


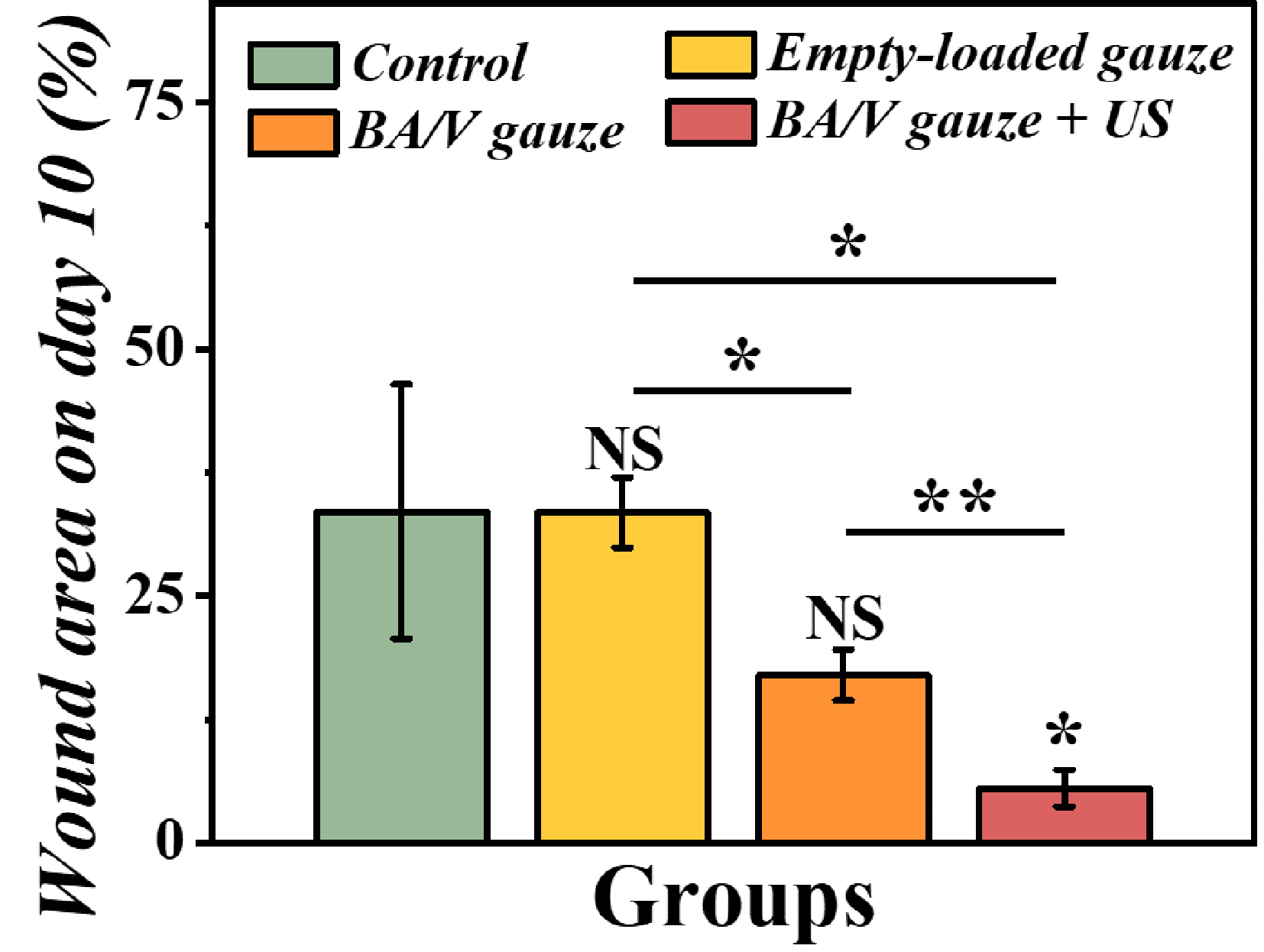


Figure S8. Statistical discrepancies of the wound areas on day 10.


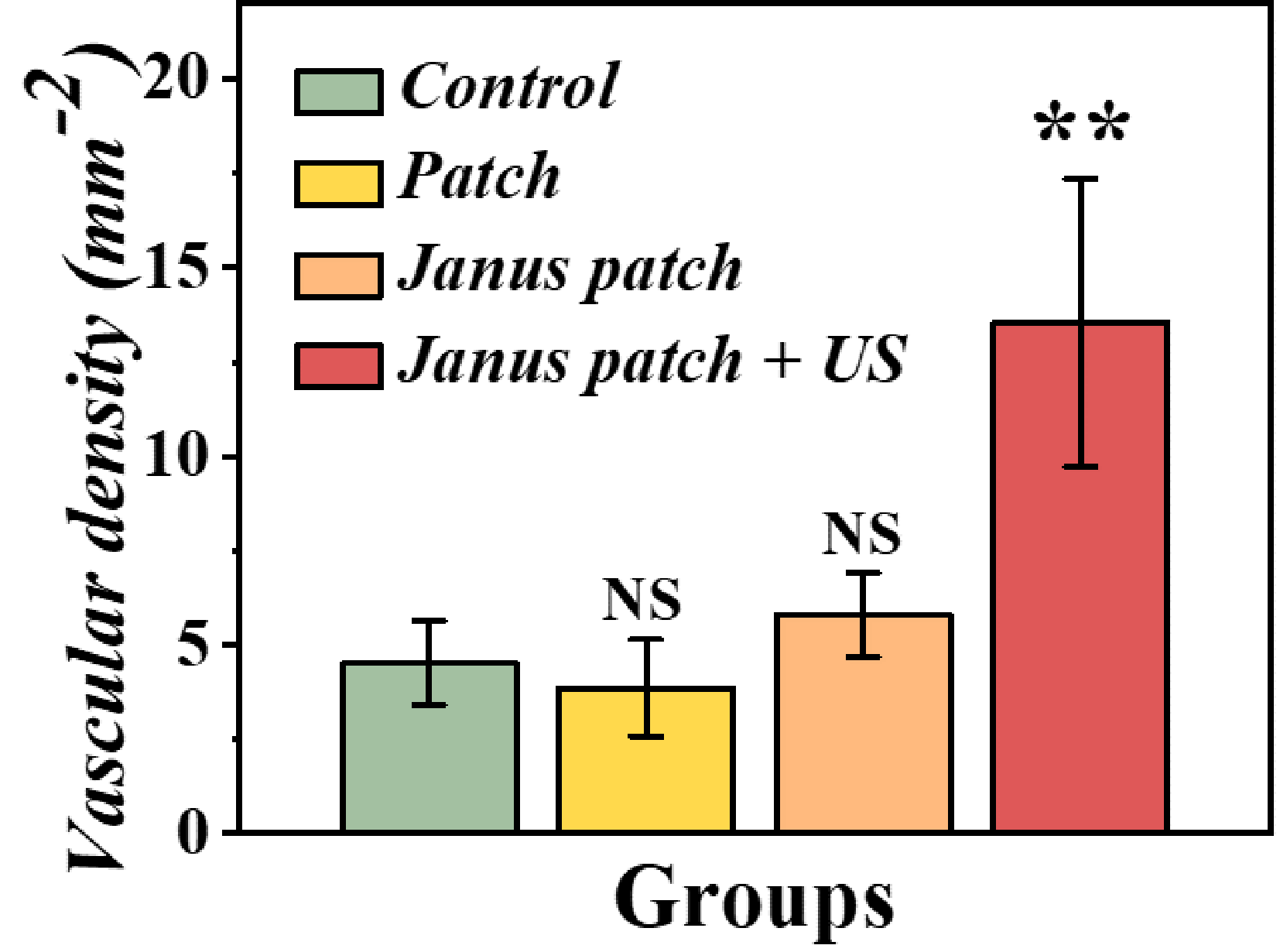


Figure S9. Quantitative study of density of blood vessels from different groups on day 10.
